# Diphtheria

**Published:** 1861

**Authors:** J. J. Morgan

**Affiliations:** Windham, Iowa


### DIPHTHERIA.

By Dr. J. J. MORGAN, of Windham, Iowa.

In attempting to write a paper upon a subject involving such a multiplied nomenclature, such a diversity of opinion; and such a variety of treatment, it is hardly to be presumed, that anything new, or more satisfactory, can be added to the many able productions that have been elicited since the prevalence of the disease in this country. I have at hand some fifteen different terms, by which the disease is professed to be known. As a choice of the numerous titles with which this affection is honored, there seems to be a preference for that of "Diphtheria," claimed to be expressive of the membranous exudation, which is a very prominent symptom, not to say an essential characteristic of the disease. So characteristic do I consider the exudation, that it is the first thing to which I direct attention, in order to make a correct diagnosis; (and contrary to some very high authority) it is to this that I look for a favorable or unfavorable prognosis. Taking into consideration in any particular case, the physical ability to withstand the effects of a severe local lesion, until its grave character can be modified. But what are the premises upon which these views are founded? In an epidemic which prevailed here during the last four months of the past year,—I was familiar with the affection from the mildest form up to those grave cases where death seems to have marked its victims at one sad stroke. The first and main thing that I wish to notice here is, that death did not occur in any single instance, where the false membrane was confined to the fauces, where the exudation did not extend to the larynges proper. But in all the fatal cases that came under my observation the laryngeal symptoms predominated. The inquiry that suggests itself is: why does not death occur in those cases where the exudation is extensively developed but confined to the fauces? Granting the supposition, (which is probably a true one), that the disease depends upon some blood poison; we are led to inquire, does the fatal result arise from the original vitiated condition of the blood, depressing the nervous centers as in malignant typhus? or are the aggravated symptoms and alarming fatality due to a materies morbi, the result of an imperfect acration of the blood consequent upon the obstruction offered to respiration by the local affection? I am led to favor the latter view of the case, based upon the experience and observation of over two hundred cases. And what I wish to maintain is, that patients die from the effects of local disease, and not from the virulence of a poison with which the blood is contaminated. That cases terminate fatally after the violence of the local affection has subsided, from debility, the result of aggravated or long protracted cases is true,—but that disease where death insidiously and quietly does its sad work, did not obtain in the epidemic that rendered desolate some happy homes in this section of country. On the contrary all that horrid train of suffering that attended asphyxia was present in all the fatal cases. But as intimated above, numerous opinions are entertained in regard to the pathology, classification, and treatment of this disease; some very high authority maintaining the identity of diphtheria and scarlatina; others contending that diphtheria and croup are one disease; some affirming that constitutional treatment should be chiefly relied upon; while remedies directed to the local affection are merely collateral, others conclude that the local treatment is the most important. The only conclusion that can be reached in the present state of information, is, that in certain epidemics the indications point to the constitution as the proper channel for interference; while in others to control the local affection offers the best chances of favorable results. I am led to grant the predominance of the constitutional symptoms, in certain epidemics over those arising from the effects of the local disease, not from any evidence presented in the epidemic that recently prevailed here,—but from the testimony of those who have observed it in a different form.

Dr. George B. Wood (Pract. of Med. vol. 1, p. 521) in speaking of pseudo-membranous iflammation of the fauces, says: "In the course of the complaint, the disposition to exudation often travels downwards, and the larynx trachea, and even bronchia become lined with the false membrane, which obstructs respiration, and often leads to fatal results. This exudation of the disease constitutes indeed its chief danger. (page 522 he says:

"When the local affection is considerable, the system is brought into sympathy, and fever is developed. (p. 524) By far the most important remedies are those addr essed immediately to the part affected. There appears to be a disposition on the part of the writers upon this disease, to hold out the idea that it is to the systemic treatment that we must look for all favorable results. The view I take of it is this; that where the constitutional symptoms are of an aggravated character, the more formidable will be the local disease, the more rapid will be its descent to the air-passages; thus seriously compromising the life of the patient. This is the experience that has fallen to my lot. All that I wish to contend for in this connection is, that the constitutional treatment is secondary in importance, to the local applications; for I believe the majority of severe cases would succumb to the local obstruction, ere the systemic remedies, were they ever so potent, could alter the casis of the blood, neutralize its poisonous properties, and send a healthy flow of the life current, to stimulate anew the depressed nervous system. That local applications do effectually arrest the disease, when used before the air-passages are too seriously implicated, I think a fair trial will abundantly prove. Symptoms,—in some cases there is pain about the angle of the jaw and ear, and slight chilliness with but little if any fever. In others there is a decided chill followed by considerable febrile excitement, a rather feeble pulse, varying in frequency from ninety to a hundred and forty per minute, total anorexia, thirst, and a general debilitated condition of the system. In still another class of cases there is no apparent indisposition, and some swelling of the glands of the neck,is the first thing that attracts notice. Now if the throat be looked into before the disease is far advanced, there will be seen upon one tonsil, and sometimes on both, the characteristic exudation. In some cases small yellow or ash-colored spots will be all that can be seen, in others the whole surface of the gland is covered, and in more aggravated cases, or those of longer duration, the whole fauces will be found to be covered from the velum palati down as far as we are able to see. In extremely aggravated cases, the morbid substance continues to exude, until a heavy coating of from one to two lines in thickness is developed. The color and consistence of the exudation varies much in different cases. If the downward progress of the false membrane is not arrested, some degree of hoarseness will be observed, but an entire suppression of the voice is rare. A peculiar barking cough is sometimes present, and is evidence of the implication of the air-passages, and consequently an unfavorable symptom. Now if the disease continues unmodified, symptoms of asphyxia cast a gloom upon any favorable issue, in which we may have indulged. The countenance is anxious, respiration is laboriously performed, and calls to its aid all the abdominal force. The patient manifests a restlessness that is painful to be seen—extends the arms upon either side,—throws the body violently from one side of the bed to the other, rises suddenly up, and may even cease to breathe, while sitting erect. Paraplegia has followed a limited number of cases under my observation. I have not any evidence of direct contagion, and conclude that if contagious at all, it is very feebly so, when the disease once prevails. I believe that a want of cleanliness is productive of more cases than would otherwise occur. The treament that I have usually adopted is as follows:

In mild cases, a portion of castor-oil with a few drops of turpentine may be given, using as a gargle a solution of nitrate silver of ten grains to the ounce. If the disease is more aggravated, an emetic may be found beneficial, followed in a few hours with some mild cathartic. Either immediately preceding the giving of the emetic, or soon after its operation, I would apply to the throat, if the coating were very thick, lunar-causic, in substance finely pulverized, touching only those parts which are covered with the exudation. If the coating is rather limited in extent and not very heavy, a solution varying from fifteen to thirty grains to the ounce may be used. The applications will rarely need to be made more than twice each day, and continue until the false membrane ceases to be formed. I usually administer chlorate-potassa in from three to ten grain doses once in from three or four hours, giving between each dose from one to four grains of quinine. I have thought vinegar steam a valuable adjuvant, enabling the patient more easily to discharge flakes of false-membrane, and any mucus that may be present. I do not hesitate to believe in the attested benefit of different preparations of iron in this disease, but have not resorted to their use. Chlorate-potassa and quinine form the basis of the constitutional treatment that I have employed with very good success. A free use of nourishment should be allowed. It may consist of ordinary food if desired by the patient; when there is anorexia, beef-tea, soups, preparations with milk, wine-whey, etc., may be as freely used as the particular case will admit. After the violence of the symptoms have subsided, and the exudation ceases to be formed, it is a good plan to use some astringent gargle to hasten the healing of the abraded surface, and guard against the re-production of the false membranec. Acetate-lead and Tannin are probably among the best. Either of these may be used in the incipient stage of the disease with marked benefit, and where the onset is not too sudden and violent, may conduct the disease to a favorable issue.

